# Editorial: Global excellence in children and health

**DOI:** 10.3389/fpubh.2023.1286481

**Published:** 2023-09-14

**Authors:** Abdulbari Bener, Ihab Tewfik, Susu M. Zughaier, Andrew S. Day

**Affiliations:** ^1^Department of Biostatistics and Medical Informatics, School of Medicine, Istanbul Medipol University, Istanbul, Türkiye; ^2^Department of Evidence for Population Health Unit, School of Epidemiology and Health Sciences, The University of Manchester, Manchester, United Kingdom; ^3^Department of Nutrition and Public Health, University of Westminster, London, United Kingdom; ^4^Department of Basic Medical Sciences, College of Medicine, QU Health, Qatar University, Doha, Qatar; ^5^Department of Paediatrics, University of Otago Christchurch, Christchurch, New Zealand

**Keywords:** screening, child, infant, diarrhea, vitamin D, vitamin A, scoliosis

## Introduction

Cooperation and collaboration within and between countries is an increasingly important component of tackling and overcoming the pressing issues impacting upon children's health and wellbeing. This Research Topic (RT) aimed to highlight advances and achievements in the health and wellbeing of children across the globe. The seven included manuscripts touched on key endemic issues of diarrhoeal illnesses, micronutrient status and child development.

## Diarrhea in children

Mortality related to acute or chronic diarrhea remains a global issue, but is especially problematic in developing countries. Almasi et al. provided a focused assessment of recent trends in diarrhea-related mortality (DRM) across the world with specific focus on geospatial aspects. The authors used World Health Organization data from 195 countries and identified high DRM in African and Asian countries. They also showed that the trend of deaths shifted in 2011 from Asian countries to those in Africa.

Sanitation is an important factor contributing to diarrhoeal illness in children. Mulatu et al. conducted a prospective cohort study that included more than 6,000 preschool children followed over six years in one region of Ethiopia. More than 41% of these children developed diarrhea during the period of observation. Risk factors for diarrheal illness in these children included lack of toilet facilities, basic water sources and distance from a water source. The authors highlighted the importance of public health measures to reduce these risk factors. These measures are likely of generic importance.

Whilst breastfeeding rates were not a focus in these reports, other work clearly shows that breastfeeding provides protection against various infections, including gastrointestinal morbidity, during the first year of life (1, 2). While promoting early and exclusive breastfeeding to increase rates may be difficult, this should be still be included as an overall strategy to reduce DRM and morbidity.

## Nutrition and micronutrients

Optimal nutrition, especially early in life, is critical in optimizing child health and growth. Mustapa Kamal Basha et al. prospectively evaluated vitamin D status in 236 mothers (early and late pregnancy) and their offspring (cord blood). Unsurprisingly, vitamin D deficiency during pregnancy was associated with cord blood levels. Whilst this study was not interventional, the authors did highlight the importance of focused public health interventions to improve vitamin D status in mothers with the objective to consequently optimize the health of their children. Other data indicate that chronic vitamin D deficiency in childhood resulting in rickets remains a major worldwide public health problem in childhood, particularly in breastfeeding infants who lack sun exposure and vitamin D supplementation (3, 4).

Another fat-soluble vitamin, vitamin A, was assessed in a large cross-sectional study conducted in China (Chen et al.). Insufficiency of vitamin A was seen in more than a third of the 2194 preschool children assessed. The rate of insufficiency increased with age and was linked with socio-economic factors and diary intake. As with vitamin D, preventative strategies are required to reduce insufficiency of this vitamin and to reduce associated adverse outcomes.

Nutritional status was shown to be a factor associated with scoliosis in a large cross-sectional study conducted in China (Zou et al.). Of the more than 45,000 school children who were screened, 3.9% were found to have scoliosis. Low weight was found to be a risk factor, along with sex and age. While micronutrient status was not assessed or considered in this study, the impact of nutritional status was apparent.

## Child development

Nutrition is also a key factor in optimizing child development. Lopez et al. reported on the association between the duration of breastfeeding and subsequent specific aspects of cognitive performance in 9,116 children aged 9–10 years. This work, which arose as part of a longitudinal study involving children across the United States, demonstrated benefits of breast feeding upon particular components of cognitive function.

The final manuscript in this Research Topic focused upon motor development in preschool children. Wang et al. undertook a bibliometric analysis to provide an assessment of trends and hotspots in research focusing on motor development in early life. Over the decade of interest, 2,583 publications were reviewed. Hot topics over this time included cognitive function, neurodevelopmental disorders and health-related fitness. The authors also identified school readiness, motor proficiency, screen time and socioeconomic status as emerging research foci. These findings highlight a number of key areas of interest for further and ongoing study.

Altogether these included reports covered important and topical aspects of child health and wellbeing. While some of the works emphasized trends in global issues (such as sanitation and diarrhoeal illness), others provide a foundation for future interventional studies (such as enhancing micronutrient status). Furthermore, these reports highlight the importance of taking a global view at aspects of child health and wellbeing.

## Author contributions

AB: Conceptualization, Data curation, Formal analysis, Investigation, Methodology, Project administration, Resources, Software, Writing—original draft, Writing—review and editing. IT: Conceptualization, Methodology, Validation, Writing—original draft, Formal analysis, Resources, Writing—review and editing. SZ: Methodology, Resources, Validation, Visualization, Writing—original draft, Writing—review and editing. AD: Conceptualization, Formal analysis, Project administration, Resources, Supervision, Validation, Visualization, Writing—original draft, Writing—review and editing.
